# SERPINA3 facilitates malignant progression and remodels tumor immune microenvironment in glioma

**DOI:** 10.1016/j.bbrep.2025.102419

**Published:** 2026-01-06

**Authors:** Wanwan Wen, Qing Zhang

**Affiliations:** a Department of Ultrasound, Beijing Friendship Hospital, Capital Medical University, Beijing, 100050, China; b Department of Neurosurgery, Beijing Tiantan Hospital, Capital Medical University, Beijing, 100070, China; c Department of Neurosurgery, Beijing Chaoyang Hospital, Capital Medical University, Beijing, 100020, China

**Keywords:** Glioblastoma, SERPINA3, Glioma-associated macrophages/microglia, Malignant progression, Tumor immune microenvironment

## Abstract

Glioblastoma (GBM) have high aggression and immunosuppression characteristics, with treatment limitation and poor clinical prognosis. Glioma-associated macrophages and microglia (GAMs) constitute the majority immune cells in the tumor microenvironment (TME). Differential molecular characteristics in GBM are related to the poor prognosis and result in therapeutic resistance. Previous research revealed that elevated SERPINA3 expression portends a dismal prognosis for glioma patients. However, the relationship between SERPINA3 and glioma malignant progression, as well as its association with GAMs infiltration, remains unclear. In this study, we explored SERPINA3 levels in different grade glioma tissues and its correlation with GAMs markers. We found that an upregulated of SERPINA3 protein expression in GBM tissues compared to low-grade gliomas. Notably, the expressions of CD68 and IBA1, markers for GAMs, were significantly increased in GBM. Furthermore, we observed a strong correlation between high levels of SERPINA3, CD68, and IBA1 with reduced survival in patients with primary gliomas. Intriguingly, within GBM tissues, we further confirmed the expression of SERPINA3 in GAMs, and that SERPINA3 expression is positively associated with CD68 and IBA1 in primary gliomas, indicating remodeling of the tumor immune microenvironment. This study provides an insight into the therapeutic strategy targeting SERPINA3 in glioma patients.

## Highlights

• Elevated SERPINA3 is associated with malignant progression and dismal outcomes.
• SERPINA3 promotes infiltration of GAMs in glioma, suggesting remodeling of TIME.
• In human glioma tissues, SERPINA3 expression is found in GAMs.
• This study provides glioma patients with novel strategy targeting SERPINA3.

## 1 Introduction

Glioblastoma (GBM) is intracranial invasion tumor and contains highly malignant and immunosuppressive tumor microenvironment (TME), along with poor prognosis [1]. Despite surgical treatment, traditional chemoradiotherapy, and emerging targeted strategies, GBM almost always recurs and has therapeutic limitation due to the remodeling of tumor immune microenvironment (TIME) and intratumoral molecular heterogeneity [2]. Glioma-associated macrophages and microglia (GAMs) are composed of peripherally derived macrophages and intracerebral microglia, which constitute the majority immune cells in the TME, thus affecting the remodeling of TIME, further accelerate tumor growth and metastasis [[3], [4], [5]]. Molecular markers affecting tumor progression, such as IDH mutation, MGMT methylation, and EGFRvIII, can be used for molecular pathological diagnosis, therapeutic selection, and prognostic evaluation in glioma patients. [[6], [7], [8], [9]]. These molecular markers are vital in managing the fate of glioma cells and have been regarded as emerging targets in many clinical trials [10,11], however, few of them are ultimately successful. Therefore, there is an urgent need to reveal the mechanism of glioma development and to find new molecular targets for glioma therapy.

Despite emerging of various molecules in glioma, the therapeutic strategies and clinical outcome for GBM patients have not been effectively improved. Serine protease inhibitor A family member 3 (SERPINA3) belongs to the serine protease inhibitor superfamily [12], which synthesized in crucial organs such as the liver, brain, and aorta, it contributes to acute phase response, inflammation response, innate immune response, and proteolysis. Previous studies have shown that SERPINA3 not only was a novel diagnostic and pharmacological target for heart failure but also connected with human atherosclerosis, abdominal aortic aneurysm, and the stabilization of the aorta [13,14]. In addition, overexpression of SERPINA3 has been also widely reported to promote tumor invasion and migration, epithelial-mesenchymal-transition, further accelerate tumor progression and evolution [[15], [16], [17]]. Although SERPINB3 has been implicated in other cancer types, its functional significance in glioma, especially regarding tumor cell invasion, remains poorly defined. Previous studies have demonstrated that SERPINA3 expression is significantly enhanced in glioma and associated with poor prognosis for glioma patients, moreover, its expression have the potential to suppress the activation of CD4^+^ T cells, monocytes and Mast cells [18,19]. However, the connection of SERPINA3 level and glioma malignant progression and the GAMs infiltration has not been previously investigated. Investigating this link is important because it may uncover novel mechanisms by which gliomas evade immune surveillance and promote malignancy, offering potential therapeutic insights.

In this study, we firstly evaluated SERPINA3 levels in clinical glioma tissues and evaluated the relationship of SERPINA3 with the glioma grade. Further the level of SERPINA3, CD68 and IBA1 with prognosis are detected in glioma patients. Moreover, the relation of SERPINA3 levels and CD68, IBA1 was explored in primary gliomas. In brief, we suggest that SERPINA3 expression contributes to glioma malignant progression and TAMs infiltration. This research provides promising insights into SERPINA3 as a novel biomarker and potential therapeutic direction in glioma patients.

## 2 Materials and methods

### 2.1 HE staining, immunohistochemistry

This work complies with all relevant ethical regulations. All human donors/patients provided informed consent and were approved by Beijing Chaoyang Hospital, Capital Medical University. No preoperative radiotherapy or chemotherapy was administered to any patient. Tumor grading adhered to the 2007 WHO classification system. For histological analysis, tumor tissues were fixed in 4 % PFA and subjected to H&E staining. Immunohistochemical staining involved overnight incubation at 4 °C with primary antibodies targeting SERPINA3, CD68, and IBA1, followed by a 50-min incubation with secondary antibody. The immunocomplex was visualized using DAB, while nuclei were counterstained with hematoxylin. Images were captured using a Leica microscope (Wetzlar, Germany).

### 2.2 Immunofluorescent staining

The sections underwent blocking with 5 % BSA for 30 min at 37 °C, followed by an overnight incubation at 4 °C with primary antibodies specific to SERPINA3, CD68, and IBA1. Subsequently, they were thoroughly washed three times with PBS and incubated for 1 h with the corresponding secondary antibody. To visualize nuclei, the sections were then incubated with DAPI for 30 min. Microscopic images were captured using a Leica microscope (Wetzlar, Germany).

### 2.3 Databases acquisition and analysis

SERPINA3, CD68, and IBA1 mRNA expression data from the public databases were downloaded (http://gliovis.bioinfo.cnio.es/). Then Survival and survminer packages were loaded in R. The Surv cutpoint function identified the optimal threshold for gene expression in CGGA and TCGA datasets, segregating samples into high and low expression groups. Kaplan–Meier curves depicted OS, and log-rank tests assessed statistical significance.

### 2.4 Statistical analysis

The Shapiro–Wilk test was utilized to examine the normality of distribution among continuous variables. The mRNA expression of SERPINA3, CD68, and IBA1 among WHO II, III and IV gliomas were compared by the Kruskal-Wallis test. Kaplan-Meier survival analysis estimated the surviving of patients with primary gliomas and was performed to calculate the corresponding surviving difference according to the categorized SERPINA3, CD68, and IBA1 mRNA expression. Pearson correlation analysis was applied to explore the relationship between SERPINA3 mRNA and CD68/IBA1 mRNA expressions, utilizing SPSS 25.0 and GraphPad Prism 8 (v9.5) for statistical evaluations.

## 3 Results

### 3.1 The high level of SERPINA3 is associated with glioma grade

**Fig. 1:**
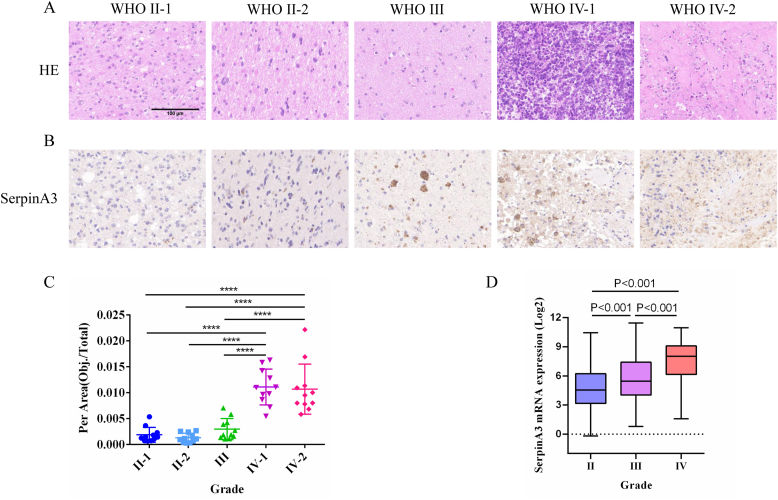
Immunohistochemical staining of SERPINA3 expression in different grade glioma tissues. A. H&E staining. B. Representative images of SERPINA3 expression (n = 3). C. The mean IOD from 5 fields/tumor section were counted. ∗p < 0.05; ∗∗p < 0.01; ∗∗∗p < 0.001; ∗∗∗∗p < 0.0001. D. mRNA of SERPINA3 from CGGA databases in different grade gliomas.

We utilized the glioma specimens to performed the related experiments. Firstly, HE

staining was performed in different grade glioma tissues (Fig. 1A). To explore SERPINA3 expression in human brain glioma tissues, we conducted immunohistochemistry staining to evaluate the levels of SERPINA3 protein from collected specimens. Our results demonstrated that SERPINA3 expression level in WHO IV glioma tissues were significantly increased compared with other WHO grade tissues (Fig. 1B and C), indicating potential malignant progression. Furthermore, we mining related data from the CGGA databases, the results displayed that the levels of SERPINA3 mRNA in WHO III and IV gliomas were higher than that of WHO II (Fig. 1D). The above data confirm that SERPINA3 expression are correlated with glioma grade and may promote the tumor malignant progression.

### 3.2 GAMs infiltration is significantly elevated in high-grade glioma tissues

**Fig. 2:**
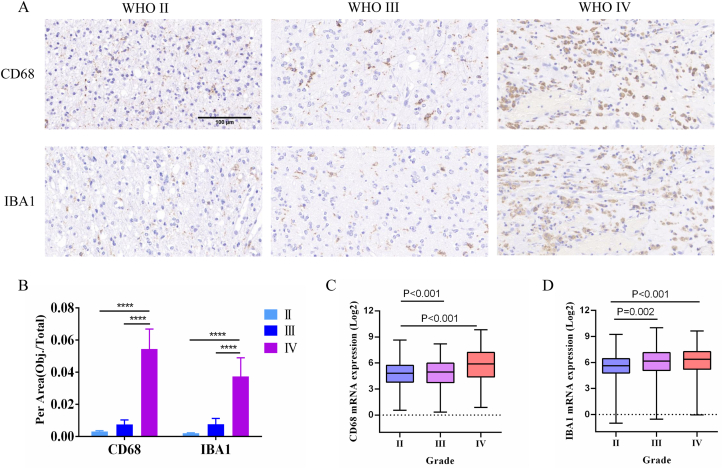
Immunohistochemical staining of CD68 and IBA1 expression in different grade glioma tissues. A. Representative images of CD68 and IBA1 expression (n = 3). B The mean IOD from 5 fields/tumor section were counted. ∗p < 0.05; ∗∗p < 0.01; ∗∗∗p < 0.001; ∗∗∗∗p < 0.0001. C-D. mRNA of CD68 and IBA1 from CGGA databases in different grade gliomas.

**Fig. 3:**
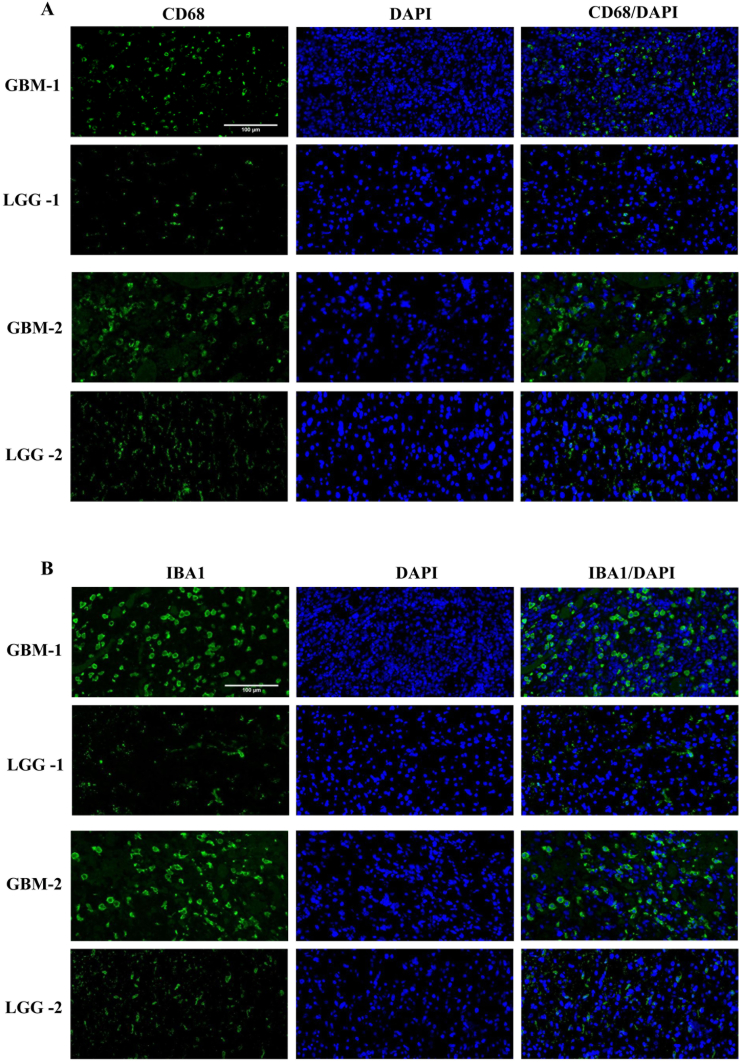
CD68 and IBA1 expression in glioma tissues (n = 3). A. Immunofluorescence staining of CD68. B. Immunofluorescence staining of IBA1.

GBM is highly immunosuppressive malignant brain tumor. Glioma-associated macrophages/microglia comprise the most population of immune cells in the TIME. To examine the GAMs infiltration in different grade gliomas. We evaluate the expression of CD68 and IBA1 protein using immunohistochemistry staining. Our data confirmed that CD68 and IBA1 expression in WHO IV glioma were significantly enhanced compared with other WHO grade gliomas (Fig. 2A and B). Moreover, we found that CD68 and IBA1 mRNA levels in WHO III and IV gliomas were higher than that of WHO II (Fig. 2C and D). We also confirmed through immunofluorescence staining that the expression of CD68 and IBA1 is higher in GBM specimens than in low-grade gliomas. (Fig. 3A and B). All in all, our studies indicated that GAMs infiltration is positively correlated with glioma malignancy grade.

### 3.3 SERPINA3, CD68 and IBA1 is associated with poor prognosis of primary glioma patients

**Fig. 4:**
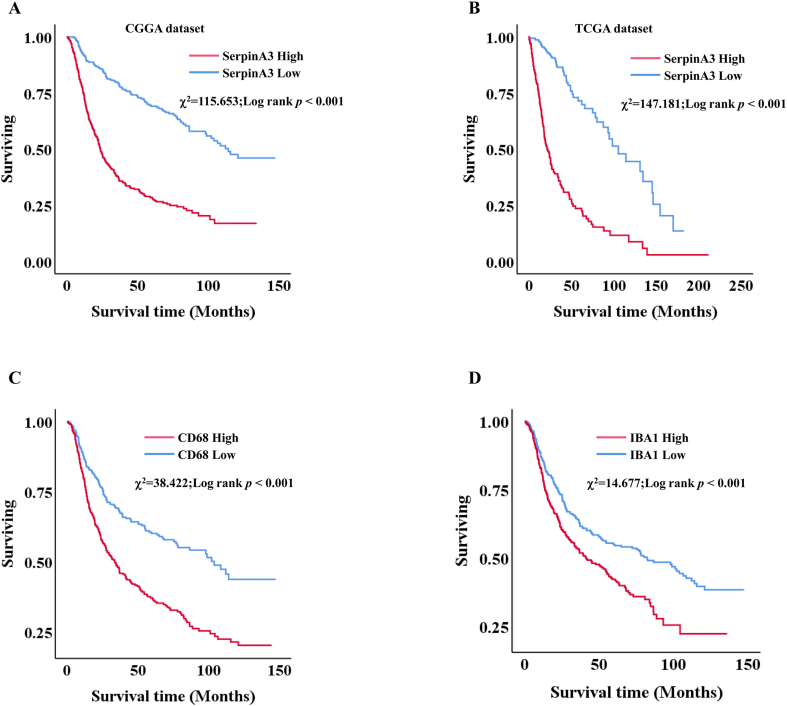
Expression of SERPINA3, CD68 and IBA1 predicted the survival for patients with primary gliomas. A. SERPINA3 in CGGA databases. B. SERPINA3 in TCGA databases. C. CD68 in CGGA databases. D. IBA1 in CGGA databases.

Despite the current multimodal treatment strategies, glioma patients have a very poor prognosis. We have performed a differential expression analysis comparing SERPINA3 and key GAM markers between glioma samples and non-tumor tissues from the databases, which confirmed significant upregulation of SERPINA3 and GAM markers in both LGG and GBM tumors compared to normal tissues (Fig. S1). To explore the molecules that influence prognosis, we further mining the survival data from the public databases about SERPINA3, CD68 and IBA1 for glioma patients. Kaplan-Meier survival analysis revealed an inverse correlation between SERPINA3 expression and prognosis in primary glioma patients from the CGGA database (Fig. 4A). This finding was subsequently confirmed in TCGA data using the same analytical approach. (Fig. 4B). In addition, we further mining the survival time of CD68 and IBA1 mRNA in glioma patients from the CGGA databases. The results displayed that high levels of CD68 mRNA have shorter surviving compared to low expression in glioma patients (Fig. 4C). Furthermore, the high expression of IBA1 mRNA for glioma patients also have a very poor prognosis (Fig. 4D). These results indicated that the high level of SERPINA3 mRNA showed a strong correlation with the dismal prognosis of patients in primary gliomas.

### 3.4 SERPINA3 expression is associated with CD68 and IBA1 in primary gliomas

**Fig. 5:**
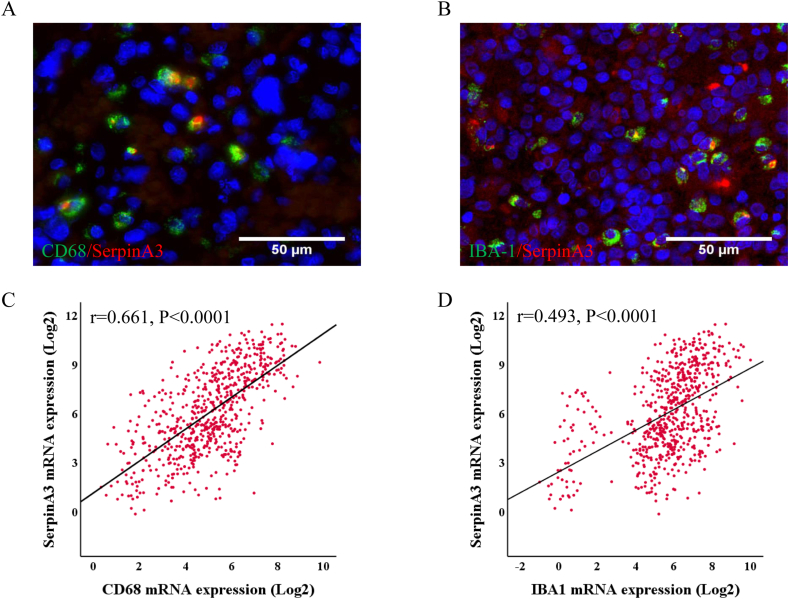
In glioma tissues, expression of SERPINA3 in macrophages and microglia. A. Immunofluorescence staining of CD68/SERPINA3 (n = 3). B. Immunofluorescence staining of IBA1/SERPINA3 (n = 3). C. Correlation of mRNA expression between SERPINA3 and CD68 in CGGA databases. D. Correlation of mRNA expression between SERPINA3 and IBA1 in CGGA databases.

**Fig. 6:**
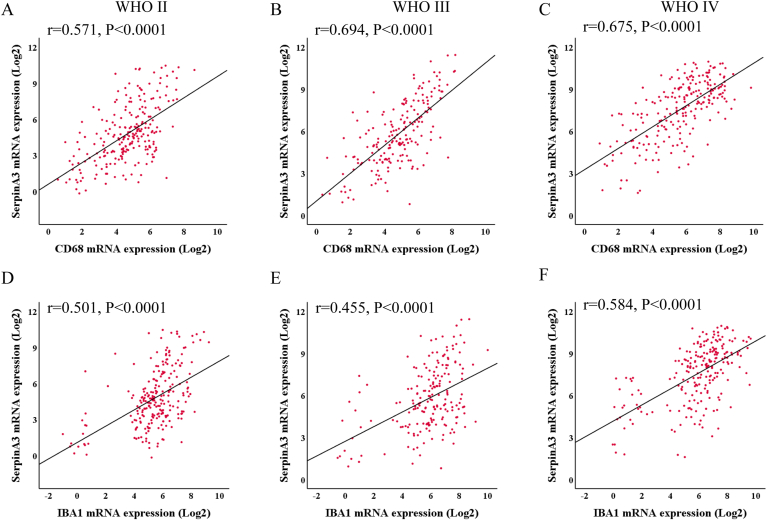
Correlation analysis of SERPINA3 with CD68 and IBA1 from CGGA databases in different grade gliomas. A-C. Correlation of SERPINA3 and CD68. D-F. Correlation of SERPINA3 and IBA1.

To explore the correlation between SERPINA3 levels and CD68, IBA1 in primary gliomas. We conducted immunofluorescence co-expression experiments using glioma specimens. The data demonstrated that co-expression of CD68/SERPINA3 and IBA1/SERPINA3 in glioma specimens, indicating SERPINA3 expression in GAMs (Fig. 5A and B). Analyzing CGGA data, we observed a positive correlation between SERPINA3 mRNA expression and the levels of CD68 and IBA1 mRNA in primary gliomas, suggesting a link between SERPINA3 and GAM markers. (Fig. 5C and D). Further, we examined the relation of SERPINA3 and these molecules in WHO II, III and IV gliomas form CGGA datasets and the mRNA level of SERPINA3 showed a positive correlation with CD68 (Fig. 6A–C). Subsequently, the relationship between SERPINA3 mRNA and IBA1 was elucidated in the data of CGGA according to the same analysis (Fig. 6D–F). SERPINA3 expression displayed a positive association with CD68 and IBA1 in primary gliomas, as evidenced by the data.

## 4 Discussion

Dismal prognosis of glioma patients can result from the highly immunosuppressive TME and malignant grade, especially GBM [[20], [21], [22]]. Despite multiple emerging treatment modes [23,24], the clinical outcomes remain to not be very optimistic. Therefore, investigating novel molecular functions and features in glioma could yield crucial insights for developing effective treatment strategies. Our study underscores the association between SERPINA3 expression and glioma grade, as well poor prognosis, it is suggested that SERPINA3 has the potential to promote glioma malignant progression. Moreover, we confirmed the co-expression CD68/SERPINA3, IBA1/SERPINA3 in glioma specimens, indicating SERPINA3 may contribute to the intratumoral infiltration of GAMs. Specifically, SERPINA3 mRNA level was found to be positively correlated with the mRNA levels of CD68 and IBA1. High levels of CD68 and IBA1 mRNA have shorter survival time compared with low levels in glioma patients. While our study provides evidence linking SERPINA3 to glioma malignancy, a key limitation is that the precise mechanistic pathways through which SERPINA3 exerts its pro-tumorigenic effects are not fully elucidated. Future studies should employ proteomic approaches and cell-specific knockout models to definitively establish the causal relationships.

An increasing body of evidence has suggested that SERPINA3 over-expression is correlated with cancer progression and dismal prognosis. Recent studies have confirmed that SERPINA3 have the potential to promote tumor invasion and migration, epithelial-mesenchymal-transition in breast cancer, hepatocellular carcinoma and lung cancer [15,25,26]. Moreover, the correlation between SERPINA3 and several related molecules are evaluated, such as MCM6, IGFBP2, and FKBP10. These molecules have been found to accelerate tumor cell proliferation and indicate the poor survival in patients [18,27,28]. Accumulating data reveal tha SERPINA3 level is associated with the adverse clinical outcomes in multiple tumors [19,29]. In our study, increased SERPINA3 expression correlates with glioma grade and poorer survival outcomes. Taken together, these results demonstrate that SERPINA3 may promote the malignant progression of glioma.

A recent study highlighted that SERPINA3 is crucial mediator in the activation, differentiation, proliferation and infiltration of immune cells and TIME reprogramming [30,31]. Previous studies have not clearly indicated SERPINB3 expression in tumor-associated macrophages or microglia, and its function within these cells remains entirely unexplored. Our observation of SERPINB3 in the glioma microenvironment now prompts this important new line of investigation. Herein, for the first time, a thorough investigation was conducted into the regulatory role of SERPINA3 in GAMs within glioma tissues. We reported that SERPINA3 have the potential to enhance GAMs infiltration in glioma specimens, further remodeling glioma immune microenvironment. Given that SERPINA3 levels were positively correlated with the expression of CD68 and IBA1 in CGGA databases, suggests that SERPINA3 may involve in forming glioma immunosuppression. Although the differential expression of SERPINA3 and GAM markers was validated using authoritative public datasets, the lack of in-house, patient-matched normal brain tissue remains a limitation in our study. Future studies incorporating these controls would provide a more rigorous foundation for comparison. The potential efficacy of immune therapy strategies in glioma, particularly those based on immune checkpoint inhibition, offers a promising avenue for treatment. Previous research revealed that SERPINA3 could potentially contribute to the PD-L1-driven immune evasion mechanisms in tumors [18]. Furthermore, tumor-associated macrophages that expressed PD-L1 suppress CD8^+^T cell responses, which lead to T cell dysfunction and ineffectiveness, further induced tumor immunosuppression [[32], [33], [34]].Despite the immunotherapy of immune checkpoint inhibition can potentiate anti-tumoral immune response in GBM [[35], [36], [37]], additional experiments are warranted to delve into the underlying mechanisms linking SERPINA3 and glioma-associated macrophage (GAM) infiltration., and perhaps a novel strategy targeting SERPINA3 and combination therapy will be developed for glioma treatment. Nonetheless, comprehensive analysis of large-scale datasets and in-depth mechanistic studies are essential to confirm the role of SERPINA3 in glioma.

## 5 Conclusions

Our study revealed that SERPINA3 is upregulated in high-grade glioma tissues and correlates with poor survival outcomes in glioma patients. Further, SERPINA3 fosters GAMs infiltration within the TME, contributing to a worse prognosis. Therefore, SERPINA3 emerges as a potential therapeutic target and prognostic marker for glioma patients.

## Consent to participate

Written informed consent was obtained from all subjects.

## Ethics approval

This study was performed in line with the principles of the Declaration of Helsinki. Approval was granted by the Beijing Chaoyang Hospital Medical Ethics Committee (No. 202322419).

## Funding

This work was supported by the 10.13039/501100001809National Natural Science Foundation of China (No. 82303422).

## CRediT authorship contribution statement

**Wanwan Wen:** Data curation, Formal analysis, Methodology, Software, Writing – original draft. **Qing Zhang:** Conceptualization, Data curation, Funding acquisition, Investigation, Supervision, Validation, Visualization, Writing – review & editing.

## Declaration of competing interest

The authors declare no conflict of interest.

## Data Availability

Data will be made available on request.
