# Supplementary material for: SERPINA3 facilitates malignant progression and remodels tumor immune microenvironment in glioma
Source: Biochem Biophys Rep. 2026 Jan 6;45:102419. doi: 10.1016/j.bbrep.2025.102419 (PMC12808515; doi:10.1016/j.bbrep.2025.102419)
Supplement: Multimedia component 1 [file mmc1.docx]

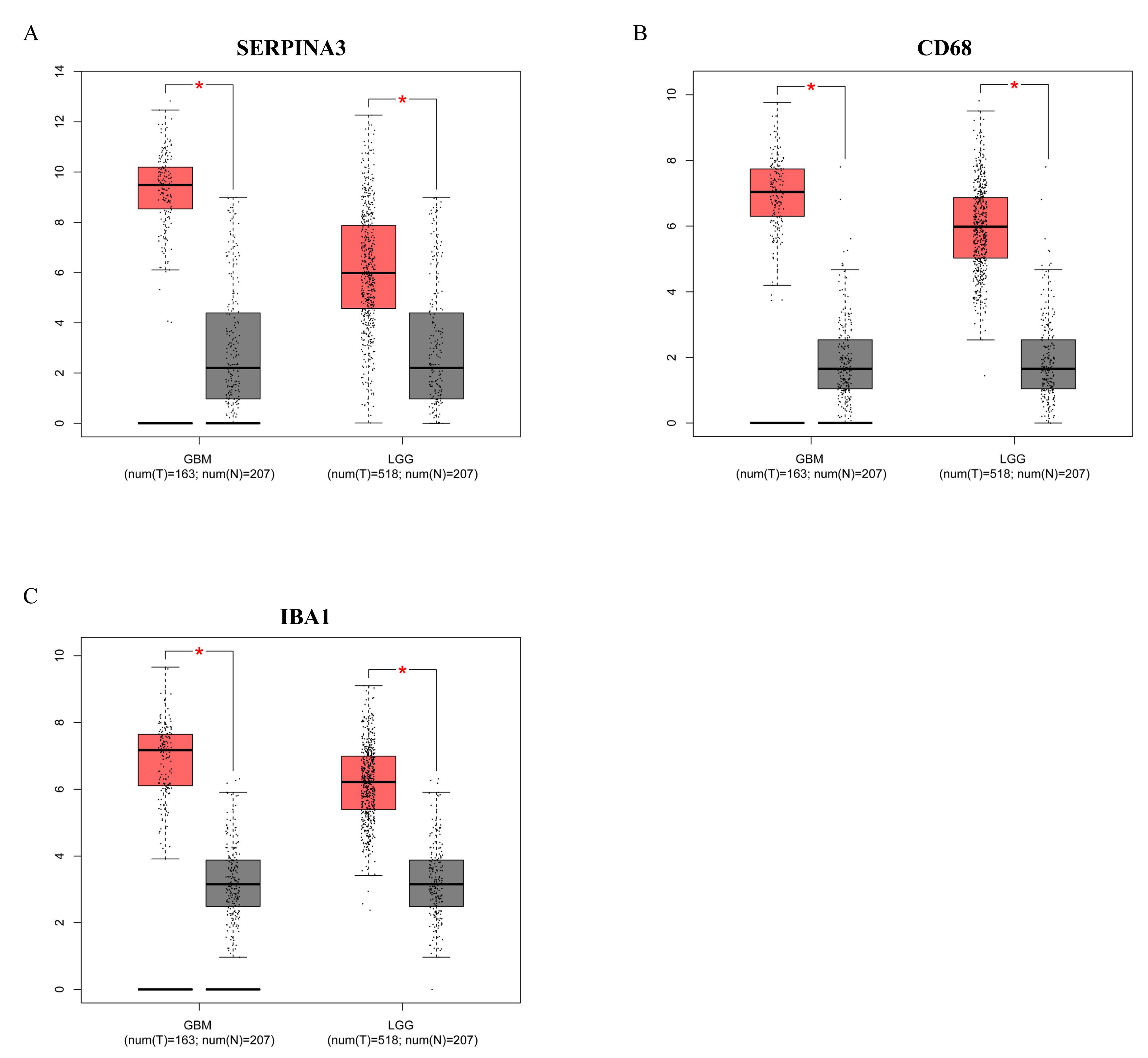


**Figure S1.** S Analysis of the GEPIA database revealed a marked upregulation of SERPINA3, CD68, and IBA1 in both LGG and GBM tumors compared to normal tissues.
